# The Effects of Chlorhexidine and Persica Mouthwashes on Colonization of *Streptococcus mutans* on Fixed Orthodontics O-rings

**Published:** 2015-03

**Authors:** Fereshteh Saffari, Mohammad Danesh Ardakani, Hengameh Zandi, Hamed Heidarzadeh, Mohammad Hassan Moshafi

**Affiliations:** a Dept. of Microbiology, School of Medicine, Kerman University of Medical Sciences, Kerman, Iran.; b Dept. of Pathology, School of Dentistry, Yazd University of Medical Sciences, Yazd, Iran.; c Dept. of Microbiology, School of Medicine, Yazd University of Medical Sciences, Yazd, Iran.; d School of Pharmacy, Kerman University of Medical Sciences, Kerman, Iran.; e Dept. of Pharmaceutics, School of Pharmacy, Kerman University of Medical Sciences, Kerman, Iran.

**Keywords:** Chlorhexidine, Mouthwash, Persica, *Streptococcus mutans*

## Abstract

**Statement of the Problem:**

Fixed orthodontic appliances predispose patients to dental caries. Use of mouthrinses has been introduced as the effective way for reducing dental plaque accumulation.

**Purpose:**

The aim of this study was to compare the effects of Persica mouthwash and Chlorhexidine (CHX) on colonization of *Streptococcus mutans* (*S. mutans*) on fixed orthodontic O-rings.

**Materials and Method:**

Thirty patients with fixed orthodontic appliances and proper oral hygiene were randomly provided by CHX and Persica and trained to use these mouthwashes according to the manufacturer’s instruction. Sampling was carried out right before and 4 weeks after mouthrinsing treatment. The mean amounts of *S. mutans* colonies in these groups were compared.

**Results:**

Comparison of *S. mutans *colonization within each group revealed both mouthrinses to be efficient. However, this difference was found to be significant only in CHX group.

**Conclusion:**

Persica cannot be a good alternative mouthwash and patients on orthodontic treatment are still recommended to use CHX.

## Introduction

Practicing satisfactory dental hygiene is one of the main subjects that orthodontic patients are encountered. Failure to achieve this goal makes these patients vulnerable to dental caries. That is because fixed orthodontic appliances inhibit effective tooth brushing and causes potential food retention, which in turn, this interferes with the oral cavity homeostasis, i.e. changes the salivary pH and the composition of the oral flora and consequently increases the dental plaque accumulation. [1-2]

Dental plaque formation is the initial step in dental caries and *Streptococcus mutans* (*S. mutans*) is considered as the main colonizer in this multi-species dental biofilm. [1] During orthodontic treatment, up to 5-fold increase in the number of *S. mutans* has been observed. [2] Both these considerations imply the need for prophylactic measures against colonization of *S.mutans*.

Although mechanical removal of dental plaque is still regarded as a promising approach, [3] high incidence of dental and gingival diseases makes the use of an adjunctive method for improving the oral health unavoidable. Use of mouthrinses has been introduced as the effective way for reducing dental plaque accumulation. [4] Among frequently used antiseptic mouthwashes, Chlorhexidine (CHX) is known as the most potent chemical agent. Currently, due to some undesirable side effects reported from CHX consumption, the tendency to use herbal mouthwashes has increased. Persica is a mouthwash containing an alcoholic extract of *Salvadora persica* (*S. persica*). Although the use of Persica is consistent with cultural and religious beliefs in our region, its oral health efficacy is still a matter of debate. [5] The aim of this study is to compare the effect of Persica mouthwash with CHX on colonization of *S.mutans* on the fixed orthodontic O-rings.

## Materials and Method

In this double-blind parallel clinical trial, 30 patients with fixed orthodontic appliances were selected from those referring to the Orthodontic Clinic in the Faculty of Dentistry at Shahid Sadoughi University of Medical Sciences, Yazd, Iran.

The study was conducted under the approval of Medical Ethical and Methodological Committees of Shahid Sadoughi University of Medical Sciences. Written informed consent was obtained from all patients included in the study.

Those who met the following inclusion criteria were enrolled in the study; orthodontic patients aged between 13-17 with proper oral hygiene (plaque index ≤ 20% measured by O’Leary index using plaque disclosing agent), [6] normal gingiva without inflammation and no detectable carious lesions. Additionally, volunteers being on antibiotic treatment, having active periodontal diseases, mouth breathing or any other problems which could affect oral microbial flora were excluded. Not having used any mouthrinses for at least 1 month before the initiation of the study, and revealing no sign of sensitivity to mouthwashes during the study were considered, as well. Regarding the oral hygiene maintenance during the treatment, all subjects were counseled by a single person.

The two studied mouthrinses were 0.2% CHX (Behsa Co., Iran) and Persica (Poursina Co., Iran). Participants were randomly given CHX and Persica and trained to use mouthwashes after tooth brushing according to the manufacturer’s instruction (15 drops in 15 ml of water for 30 seconds, twice a day). Sampling was performed in two steps: the first step four weeks after bonding and the second one, 4 weeks following the mouthrinse treatment.  All of the microbial tests were carried out in the microbiology laboratory of Medical Faculty of Shahid Sadoughi University of Medical Sciences. Briefly, the elastic O-rings were removed and collected in sterile normal saline solution. After immediate transfer to laboratory, samples were homogenized and then 10-fold serial dilutions (10 ^-1^-10 ^-5^) were prepared using phosphate-buffered saline solution (PBS). Next, 0.01 ml of each diluent was inoculated on to the Mitis Salivarius Agar medium containing 0.001% potassium tellurite (MSAT) (Merck, Germany). After 48 hours of incubation at 37°C in 10% CO2, the number of *S.mutans* colonies was determined. [4, 7] Each test was performed in triplicate and finally the mean value was reported as colony forming unit (CFU). The biochemical tests for identification of *S.mutans* included catalase, hydrolysis of esculin and arginine, and fermentation of carbohydrates; mannitol and sorbitol.

Statistical analysis was performed using IBM SPSS Statistics version 21. For all data analysis, *p* < 0.05 was considered significant. Non-parametric data were examined using Mann-Whitney U-test. Comparison analysis of colonization pre- and post-mouthrinsing within each group was performed using the Wilcoxon signed-rank test.

## Results

A total of 30 patients with fixed orthodontic appliances (mean age: 14.6±1.2) having similar condition in terms of general oral hygiene, were instructed thoroughly to use CHX and Persica for one month. Mouthwashes were randomly distributed, independent of sex and age.

The mean values of *S. mutans* colonies in both groups, preceding mouthrinses application, were similar (Table 1). Comparison of *S. mutans* colonization within each group, showed the reduction effect of both mouthrinses. However this difference was significant only in CHX group (Table 1).

**Table 1:** Comparison of *S. mutans* colonization on orthodontic O-rings between two groups

**Group (No)**	**Mean value CFU ± S.D** **Pre-mouthwash treatment**	**Mean value CFU ± S.D** **Post-mouthwash treatment**	**P-value**
CHX	18.9 ± 10.7	1.4 ± 1.8	0.001
Persica	19.2 ± 10.6	14.8 ± 2.1	0.86

S.D: standard deviation

## Discussion

Fixed orthodontic appliances predispose patients to plaque accumulation around brackets and gingival margins. As mechanical tools seem to be not enough in controlling dental plaque in these patients, application of mouthwashes has been suggested as the supplement of oral hygiene practices. [3, 8]

CHX is a cationic mouthwash with the highest oral health effects among antiseptic mouthwashes which the efficacy of all other components is compared to. [9] However, the associated shortcomings such as unpleasant taste, tooth staining and irritation of oral mucosa have encouraged the researchers to find appropriate alternatives. Persica is a herbal mouthwash composed of alcoholic extract of the following plants, *S. persica* (30%), *Millefolium Achillea* (25%) and spearmint (45%). [3, 9]

As it is obvious in table 1, no significant difference was found between the two groups prior to using mouthwashes which shows that both groups are similar regarding the status of *S. mutans* colonization. In the present study, significant difference was found in *S.mutans* colonization between the first and second steps within CHX group (*p*= 0.001). On the other side, slight reduction was seen in the mean count of colonies following Persica treatment which was not significant compared to the pre-treatment values (*p*= 0.86). Comparison of colonization between the two groups, following mouthrinse treatment revealed CHX to be significantly more effective than Persica (*p*< 0.05).

The effect of CHX as the most potent agent has been supported by many studies; Enita *et al.* reported that CHX-based mouthwashes reduced *S. mutans* counts and improved the gingival index in orthodontic patients. [1] Similarly, Olympio *et al.* suggested that use of CHX-containing dentifrices seems to be effective for the treatment of gingivitis. [10] Considering the limitations of CHX usage, some researchers have demonstrated that dentifrices with lower concentration of CHX can reduce the risk of tooth staining without compromising its effectiveness in controlling gingivitis and bleeding in orthodontic patients. [11] Additionally, *in vitro* comparison of antimicrobial effects of CHX, Persica and Miswak extract by Moeintaghavi *et al.*, introduced CHX as the most effective antibacterial agent. [9] In contrast, Attin *et al.* found that application of a highly concentrated CHX varnish in patients with fixed orthodontic appliances does not result in a distinct reduction of *S. mutans* colonization. [12] Albeit, the weak statistically significance met in that study can be attributed to differences in sampling.

Notably, the benefit of Persica mouth rinse in maintaining oral health is questionable. [7, 9] Our findings appear to be in contrast with the study of Khalessi *et al.* in which *in vivo* study of plaque control efficacy by Persica showed significant decrease in the carriage rate of cariogenic bacteria. [5] We believe that this may be related to different participants enrolled; patients with fixed orthodontic appliances versus healthy volunteers. Likewise, Salehi *et al.* demonstrated a comparable antimicrobial effect of Persica to that of CHX and so, they recommended Persica because it was not associated with undesirable side effects. [7] Differences between the participants, particularly in nutrition habitat and cultural conditions may be involved in obtaining results different from the current study. In study performed by Singh *et al.*, it was found that CHX was the most potent in preventing plaque regrowth compared to a newly formulated herbal mouthrinse; HiOra. [3] One of the primary constituents of HiOra is *S. persica*. In another study, Rahmani *et al.* assessed the effects of this component as well as CHX on plaque formation and found a comparable plaque inhibitory by both solutions. [13] Additionally, strong *in vitro* antibacterial effect of Miswak - from which *S. persica* is extracted- against oral microorganisms associated with periodontitis and caries, was disclosed by Sofrata *et al.* [14]

## Conclusion

According to this study, Persica cannot be a good alternative for CHX. However, regarding the favorable taste and other aforementioned beneficial oral health effects, a larger long-term clinical trial is needed. More prolonged exposure is an option that may improve its efficacy. Combining Persica components with other known antiseptic agents or developing new formulations are also suggested. After all, inhibition of bacterial colonization on the orthodontic o-rings by using mouth rinses such as CHX will help orthodontic patients improve their periodontal health during treatment. 
